# Evaluation of the synergistic impact of needle and forceps biopsy with electromagnetic navigation bronchoscopy: the CONFIDENT-ENB trial design

**DOI:** 10.1186/s12890-022-02104-w

**Published:** 2022-08-19

**Authors:** Yeon Wook Kim, Hyung-Jun Kim, Sung Hyun Yoon, Kyung Hee Lee, Young Mi Park, So Yeon Ahn, Myung Jin Song, Byoung Soo Kwon, Sung Yoon Lim, Yeon Joo Lee, Jong Sun Park, Young-Jae Cho, Ho Il Yoon, Jae Ho Lee, Choon-Taek Lee

**Affiliations:** 1 Division of Pulmonary and Critical Care Medicine, Department of Internal Medicine, Seoul National University Bundang Hospital, 173-82 Gumi-Ro, Bundang-gu, Seongnam, 13620 Republic of Korea; 2 Department of Radiology, Seoul National University Bundang Hospital, Seongnam, Republic of Korea; 3 Medical Research Collaborating Center, Seoul National University Bundang Hospital, Seongnam, Republic of Korea

**Keywords:** Electromagnetic navigation bronchoscopy, Lung neoplasms, Diagnostic imaging, Pulmonary medicine

## Abstract

**Background:**

Electromagnetic navigation bronchoscopy (ENB) is an emerging advanced imaging-guided bronchoscopy technique for diagnosing peripheral lung lesions. However, the selection strategy for the optimal biopsy device and whether adopting a multi-tool strategy increases the diagnostic yield remains undetermined. The CONFIDENT-ENB trial (NCT05110131) is a prospective randomized study on ENB, performed in a least-invasive setting. The primary aim is to evaluate whether a combination of needle aspiration and forceps biopsy improves the diagnostic performance, and assess the comparative diagnostic value and discordance of the two devices.

**Methods:**

The trial will recruit 142 participants with lung lesions suspected of malignancy who are eligible for an elective ENB procedure under moderate sedation. Participants will undergo ENB-guided needle aspiration and forceps biopsy in a randomized order without the use of any complementary techniques. All participants will be followed up subsequently for up to 12 months to conclude the final diagnosis of the biopsied lesions. Primary outcomes include the diagnostic yield and sensitivity of each biopsy modality and the diagnostic yield of the combined modalities.

**Discussion:**

The CONFIDENT-ENB trial will prospectively evaluate the synergistic effectiveness and comparative accuracy of ENB-guided needle aspiration and forceps biopsy in a least-invasive setting. The results are expected to improve our understanding of the optimal tool-selection strategy for ENB.

*Trial registration*: ClinicalTrials.gov (NCT05110131). Prospectively registered on 5 November 2021.

## Background

Lung cancer remains the common most cause of cancer-related deaths among both men and women globally [1]. Early and accurate diagnosis is important to facilitate curative intent. Recently, widespread efforts for early detection and implementation of lung cancer screening with low-dose chest computed tomography (CT) have resulted in a considerable increase in the identification of peripheral lung lesions, suspected of being lung cancer, which require diagnostic evaluation [2–4]. The histopathological diagnosis of pulmonary lesions is a challenging process that has been attempted using various techniques. According to current clinical practice guidelines, the least invasive approach with the highest yield is recommended [5–7]. For peripheral lesions that are difficult to reach with conventional bronchoscopy, transthoracic needle aspiration (TTNA) is commonly used. However, despite a good sensitivity of approximately 90%, TTNA carries a reported pneumothorax rate of > 18% of the biopsied cases [8, 9].

Recently, in an attempt to improve the yield and safety of flexible bronchoscopy, several image-guided technologies have been developed [10]. Emerging bronchoscopy techniques include electromagnetic navigation bronchoscopy (ENB), which involves generating an electromagnetic field around the patient. This technique allows physicians to access peripheral lung lesions through a minimally invasive technique using an image-guided flexible catheter and a dedicated navigation software system. Although the reported diagnostic performance of ENB varies between studies, it is fairly accepted to yield good accuracy in diagnosing malignancy with a yield of over 70% and a markedly lower complication rate (< 3% for pneumothorax) than TTNA [11–13]. According to current guidelines, ENB is recommended for peripheral lung lesions beyond the reach of conventional flexible bronchoscopy alone [4, 5].

For diagnostic bronchoscopic procedures, including ENB, the most commonly used sampling methods are forceps biopsy and needle aspiration [14, 15]. Previous studies suggest that the diagnostic yield may be related to the choice of biopsy tool [16–18]. Particularly, several studies have reported a better diagnostic yield with the aspirating needle than with other transbronchial techniques [16, 17, 19, 20]. Furthermore, a few studies suggest that the use of a multi-tool strategy can improve the diagnostic yield [19, 21, 22]. However, multiple studies that have published real-world data on ENB have reported the use of a single-tool strategy with low use of the aspirating needle, which may have contributed to the overall low diagnostic yield of ENB [16, 23–25]. A disparity exists in the context of a higher reported diagnostic yield with the aspiration needle and the actual use in clinical practice. Therefore, the synergistic benefits of needle aspiration and forceps biopsy possibly derived from the relative accuracy and discordance of needle aspiration and forceps biopsy with ENB warrant further examination in a prospective, controlled study. The CONFIDENT-ENB (“COmparison and synergistic evaluation of Needle aspiration and Forceps bIopsy for Diagnosing pulmonary lEsioNs wiTh Electromagnetic Navigation Bronchoscopy”) trial is a prospective trial that aims to evaluate whether combining ENB-guided needle aspiration and forceps biopsy provides synergistic benefits, with comparison of the diagnostic performance of the two methods.

## Methods/design

### Study design and subject eligibility

**Fig. 1:**
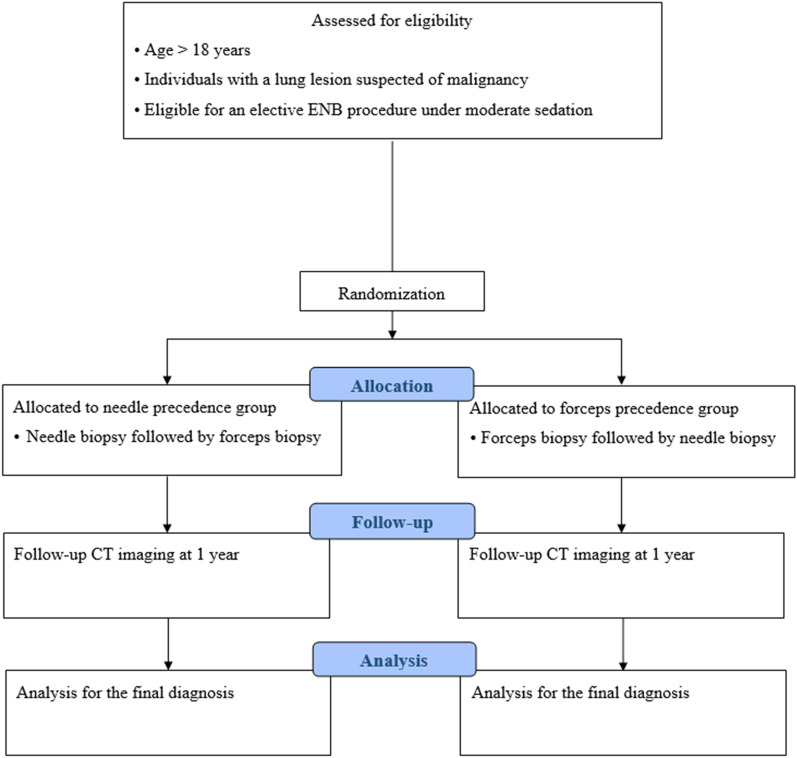
**A** Schematic figure depicting the study design of the CONFIDENT-ENB trial. ENB, electromagnetic navigation bronchoscopy; CT, computed tomography; CONFIDENT-ENB, COmparison and synergistic evaluation of Needle aspiration and Forceps bIopsy for Diagnosing pulmonary lEsioNs wiTh Electromagnetic Navigation Bronchoscopy

We designed a prospective, randomized trial to evaluate the synergistic diagnostic yield of ENB-guided needle aspiration biopsy and ENB-guided forceps biopsy by performing biopsy with both the devices in a random order (Fig. 1). This study aims to enroll approximately 142 participants from a tertiary center in South Korea by 11 pulmonary physicians. All consecutive patients over the age of 18 years who are candidates for an elective ENB procedure for histopathological evaluation of peripheral lung lesions suspected of malignancy but predicted to be unreachable with conventional bronchoscopy alone are eligible for enrollment. The exclusion criteria are: (1) patients unable or unwilling to provide informed consent or comply with the follow-up schedule; (2) patients who have participated in an investigational drug or device research study within 90 days of enrollment, with possible interference with this study; (3) patients with a history of intolerance to moderate sedation or allergies to any of the sedatives planned to use, those with comorbidities contraindicating the bronchoscopy procedure, those on mechanical ventilation or who underwent a tracheostomy, or those who are pregnant; (4) patients with multiple lesions that need to be sampled with ENB-guidance; and (5) patients who were initially planned to undergo ENB but successfully underwent biopsy from a visible endobronchial tumor lesion, which precluded the need for electromagnetic navigation, or those who failed to perform ENB-guided biopsy with both modalities.

This study is being conducted in accordance with the Declaration of Helsinki and all regulatory requirements. The protocol was approved by the institutional review board of Seoul National University Bundang Hospital (IRB no: B-2112-715-302), which is where patient enrollment and ENB procedures will be conducted. Informed consent will be obtained from all participants. The study is registered at ClinicalTrials.gov (NCT05110131). The results will be reported according to the Standards for Reporting Diagnostic Accuracy Studies (STARD) and the Consolidated Standards of Reporting Trials (CONSORT) statements.

### Randomization and baseline evaluation

After providing informed consent, the participants will be randomly allocated to a study group (needle precedence group or forceps precedence group). They will initially undergo ENB-guided biopsy with the modality of the allocated group (needle or forceps) followed by a biopsy with the other modality. Randomization will be performed by an independent statistician using a computer-generated list using SAS version 9.4 (SAS Institute, Cary, NC, USA). The attending physicians have no role in the assignment process and will be blinded to the allocation. The physicians who will perform the ENB procedure are also blinded to the allocation until the preparation of ENB on the day of the procedure.

The enrolled participants will be evaluated for demographic, clinical, and radiological characteristics at baseline (within 30 days of the procedure) and on the procedure day. The complete list of baseline demographic data and lesion characteristics evaluated with the pre-procedural chest CT is listed in Table 1. Additionally, the pre-procedural probability of malignancy of the lesion will be evaluated for each lesion using the Brock model [3].

**Table 1:** Basic characteristics of the participants

Demographics
Age (years)
Sex
Race
Smoking status
Pack-years smoked
Family history of lung cancer
Clinical characteristics
Performance status
History of lung cancer diagnosis
History of malignancies other than lung cancer
Prior invasive lung procedures and surgery
Radiological characteristics
Lesion location (lung lobe)
Lesion type (solidity)
Lesion size
Size of solid proportion
Lesion shape (speculated margin, cavity formation, bubble lucency)
Pre-procedural probability of malignancy
Distance from lesion to pleura
Emphysema surrounding lesion
Interstitial fibrosis surrounding lesion
Bronchiectasis surrounding lesion
Presence and type of bronchus sign
Combined lymphadenopathy
Positivity on positron emission tomography (if applicable)
Procedural characteristics
Preceded modality
Capability to navigate and localize the lesion
Number of biopsy attempts (for each modality)
Acquisition of a core tissue available for histologic examination (for each modality)
Procedure duration
Sequential linear EBUS for nodal staging
Total dose of sedatives used

*EBUS* Endobronchial ultrasound

### Procedures

ENB procedures will be performed using the Spin Thoracic Navigation System (SYS-4230 K; Veran Medical, St. Louis, MO, USA) by one of nine pulmonologists (HIY, YJC, JSP, YJL, SYL, BSK, YWK, MJS, and HJK, each having at least 3 years of experience with bronchoscopic procedures). On the day of the procedure, the patients will undergo inspiration/expiration chest CT before the procedure for the correlation with the navigation platforms and reconstruct virtual airway routes. The ENB procedures will be performed according to the product instructions under moderate sedation with intravenous administration of 2–3 mg midazolam and 25–50 µg fentanyl at the procedure onset. The performing physician may administer additional midazolam or fentanyl during the procedure to achieve adequate sedation. A bronchoscope with an outer diameter of 5.9 mm (BF-1TQ290), 4.9 mm (BF-260), or 4.0 mm (BF-P260F) (all Olympus Corporation, Tokyo, Japan) is used for ENB procedures. According to the allocated group, ENB-guided biopsy will be first attempted at least three times with a 22 G or 21 G aspirating needle or forceps followed by the same procedure with the other modality with at least three attempts. There are no protocol-specific restrictions on the maximum attempts and the number of attempts is subjective to the performing physician’s discretion. Successful localization and approach to the lesion, number of biopsy attempts, total procedure time, and successful acquisition of a core tissue for histological examination for each modality will be recorded.

Following biopsy using both the methods, bronchoalveolar lavage (BAL) will be performed by placing the bronchoscope into the lung segment in which the biopsy was performed. After wedging, 30 mL sterile normal saline will be instilled followed by the recovery of the specimen in a sterile container. If the retrieved lavage fluid is insufficient for cytological examination, the performing physician may additionally perform BAL. All ENB procedures will be controlled and will not include any other complementary imaging techniques, such as radial endobronchial ultrasonography (EBUS), fluoroscopy, and rapid on-site examination (ROSE). Lymph node staging using linear EBUS may be performed after the ENB procedure at the discretion of the attending physician.

Using the ENB procedure, biopsy samples from the needle and forceps biopsies, cytology samples from needle aspiration, and cytology samples from BAL are expected from each participant. All samples will be evaluated by experienced pulmonary pathologists who are blinded to the results of other sampling methods and clinical information of the patient.

### Diagnosis and follow-up

All cases with histopathologically confirmed malignancy based on either ENB-guided needle biopsy or cytology, forceps biopsy, or BAL cytology are determined as positive for malignancy. The histopathological results of the biopsied samples that reveal no malignancy or indeterminate findings are initially considered negative. In such cases, the attending physician will conduct follow-up for at least 12 months and make decisions to perform additional invasive procedures to determine the true diagnosis (gold standard) accordingly. The final diagnosis will be determined at 12 months from the procedure. The following cases will be defined as false negatives: (1) malignancy based on a repeat biopsy (e.g., surgical biopsy, CT-guided TTNA, and bronchoscopic); (2) growth of the lesion observed on serial follow-up CT within 12 months; (3) treated as lung cancer without pathological confirmation; and (4) lung cancer diagnosed at other sites (including non-index lesions or lymph nodes diagnosed using linear EBUS during or after the index procedure) within 6 months. The following cases will be defined as true negatives: (1) subsequent diagnostic procedures confirm a non-malignant diagnosis; (2) an initial non-malignant lesion resolved without cancer treatment (e.g., improved proven infectious disease or transient inflammation); or (3) no lesion progression observed on radiographic follow-up for 12 months.

### Objectives and endpoints

The primary endpoint is the diagnostic performance of ENB-guided needle biopsy and forceps biopsy and the yield achieved by combining the modalities based on the 12-month clinical follow-up. Secondary endpoints include a comparison of successful approach to the lesion, duration of sampling time, number of attempts, and successful acquisition of core tissue between the needle and forceps biopsy techniques. Additionally, all adverse events related to the ENB procedure or sedatives will be captured and classified according to the Common Terminology Criteria for Adverse Events (CTCAE) scale, version 5.0 [26]. Pneumothorax will be confirmed using chest X-rays performed post-procedurally and the day after the procedure. All participants will be followed up at 2 weeks post-procedure to evaluate the presence of delayed complications. The detailed definitions of outcomes are presented in Table 2.

**Table 2:** Definitions and overview of study endpoints

Primary endpoint: diagnostic values
- Primary endpoints will be calculated separately for needle aspiration, forceps biopsy, and the combination of the two. For initially negative results, the final diagnosis will be confirmed based on the 12 months of clinical follow-up
- Diagnostic accuracy:
Proportion of subjects in whom the ENB-guided biopsy yielded a definite diagnosis = (malignant and benign diagnosis by modality)/(biopsied lung lesions)
- Sensitivity:
= (Malignancy confirmed by modality)/(Number of malignancies confirmed at 12-month follow-up)
- Specificity:
= (Benign confirmed by modality)/(Number of benign lesions confirmed at 12-month follow-up)
- Positive predictive value:
= (True malignant lesions)/(Number of malignant lesions by modality)
- Negative predictive value:
= (True benign lesions)/(Number of benign lesions by modality)
**Secondary endpoints**
- Secondary outcomes will be calculated separately for needle aspiration and forceps biopsy
- Navigation success:
Target lesion successfully reached with the biopsy tool; identified by the dedicated navigation software
- Procedure time:
Duration from the initial introduction of the tool to the final removal of the biopsy tool
- Time per biopsy attempt:
Procedure time divided by the number of biopsy attempts
- Successful acquisition of core tissue:
Acquisition of a fresh tissue available for histological examination
Procedure-related adverse events
-Pneumothorax
Grade 1: asymptomatic; clinical or diagnostic observations only; intervention not indicated
Grade 2: symptomatic; intervention indicated (e.g., thoracic tube insertion without pleurodesis)
Grade 3: pleurodesis and/or operative intervention indicated; hospitalization indicated
Grade 4: life-threatening consequences; urgent intervention indicated
Grade 5: death
Bronchopulmonary hemorrhage
Grade 1: mild symptoms: intervention not indicated
Grade 2: moderate symptoms: invasive intervention not indicated
Grade 3: transfusion indicated; invasive intervention indicated; hospitalization
Grade 4: life-threatening consequences; intubation or urgent intervention indicated
Grade 5: death
Respiratory failure:
Life-threatening situations with urgent intubation or ventilator support indicated

*ENB-guided biopsy* Electromagnetic navigation bronchoscopy-guided biopsy

### Primary statistical plan and sample size considerations

The sample size was calculated in order to determine a 15% difference in the diagnostic yield between the individual modalities and their combined use owing to the expected better and synergistic diagnostic yield. The estimated difference in the diagnostic yield (67% vs. 82%) is based on previously conducted large-scaled observational studies and meta-analyses that suggested an improved diagnostic yield with the use of needle aspiration and a combination of both modalities [11, 12, 14, 16]. The calculation was based on this difference in the diagnostic accuracy for equality of paired proportions with a two-sided significance level of 0.05 and 80% power. Assuming that the response with needle biopsy is independent of the response with forceps biopsy, the percentage of participants with discordant results for the two modalities was estimated to be 39%. With an estimated dropout rate of 5% due to the loss of patients during follow-up, we estimated a sample size of 142 participants (71 in each group, and 142 biopsies for each modality and combination). The sample size was calculated using PASS 2021 (NCSS, LLC., Kaysville, UT, USA).

Baseline demographic, clinical, and radiological characteristics of the participants as well as the procedural details and outcomes will be presented as means with standard deviation (SD) for continuous data and frequencies with proportions for categorical data. Characteristics will be analyzed using the *t*-test (or Wilcoxon’s rank-sum test) for continuous variables and the chi-square test (or Fisher’s exact test) for categorical variables. Since each patient will have two results from separate modalities, it should be considered that two outcomes from the same patient are likely to be more similar than two results from different patients. To compare the paired proportions, McNemar’s test will be used. A *p*-value < 0.05 indicates statistical significance. All analyses will be performed using STATA version 16.0 (StataCorp., College Station, TX, USA). A preliminary analysis is prespecified when all the enrolled participants undergo ENB with available initial pathological reports and 1-month follow-up.

### Trial status

Enrollment began in December 2021 and is currently in progress with the aim of completing enrollment by mid-2023. Analysis following initial procedural results is planned for end-2023. Completion of 12 months of follow-up with the final collection of data is expected in 2024.

## Discussion

The choice of the biopsy method for diagnosing pulmonary lesions suspected of malignancy should be based on the location, size, invasiveness of the procedure, and the risk of possible complications [4]. For peripheral lesions that are difficult to reach with conventional bronchoscopy, the options include TTNA or guided bronchoscopy. Among the available techniques, the least invasive method with the highest expected yield should be preferred [6, 7]. While TTNA was traditionally preferred due to its high diagnostic yield, its complications are not rare, and the possible risk of pleural recurrence must be considered [27, 28]. Meanwhile, recent advances in image-guided bronchoscopy technologies, including ENB, radial EBUS, and virtual bronchoscopy, have demonstrated high diagnostic yield and safety, which have rendered the transbronchial approach more feasible [29, 30]. Among the advanced guided-bronchoscopy techniques, ENB was evaluated in various studies with most studies reporting a diagnostic yield of 67–84% [31, 32]. Recent meta-analyses have demonstrated that ENB-guided diagnosis provides good accuracy in diagnosing malignancies with a yield of approximately 75% and a procedural complication rate lower than 3% for pneumothorax [12]. Previous studies have revealed that the use of complementary techniques such as radial EBUS, fluoroscopy, and ROSE and the method of anesthesia are not associated with better diagnostic yield [11, 12]. These findings indicate that ENB alone without general anesthesia can achieve a good diagnostic performance and safety profiles in diagnosing peripheral lung lesions, which would be the least-invasive setting for a guided-bronchoscopy procedure that is accessible to the maximum number of physicians [33].

When performing ENB, the decision to choose the relevant biopsy tool(s) is important. Several studies have suggested that transbronchial needle aspiration (TBNA) can increase the diagnostic yield of conventional bronchoscopy and guided bronchoscopy [16, 18, 20, 34]. However, TBNA also includes limitations in approaching lesions in the upper lobes or superior segment of the lower lobes because the needle may not be able to pass through sharp angles. Despite the possible benefits, even in expert centers, TBNA is underused in real-world practice [15, 16, 23]. Similarly, for ENB, transbronchial forceps biopsy remains the mainstream modality for tissue sampling followed by needle aspiration [14, 24, 25]. Several studies have suggested a higher diagnostic yield with ENB-guided TBNA than forceps biopsy, while few studies have suggested that using multiple tools may be more effective than single-tool strategies [11, 12, 19]. When performing ENB, multiple factors such as its usage patterns, market availability, and reimbursement factors influence the choice of the biopsy tool other than the expected efficacy of any individual tool or combinations of multiple tools. Especially, the cost of using an additional tool is an important limiting factor in choosing a multi-tool strategy without clear benefits. Therefore, relevant comparisons are required between the diagnostic performances of forceps and aspirating needle biopsy, which are the two modalities that are expected to provide the highest efficacy and are most commonly used in clinical practice. Furthermore, data regarding whether a combination of the two modalities would have synergistic effects on the overall diagnostic yield would be particularly important. These needs were the essential driving forces behind the planning and design of the current trial. To minimize possible biases and inconclusiveness resulting from the use of other complementary imaging or biopsy tools and different sedation methods, we designed this study such that the procedure can be performed in a least-invasive setting under moderate sedation without the use of additional techniques other than ENB. Our study settings reflect the target circumstances of minimal invasiveness in diagnosing lung lesions using guided bronchoscopy. Furthermore, our design reflects the setting accessible to the maximum number of physicians who perform ENB in clinical settings with limited access to general anesthesia or other techniques, such as ROSE. The navigation system used for ENB will also be controlled and unified into the Spin Thoracic Navigation System.

The major limitation of our study design will be the inclusion of participants from a single center comprising only Asian ethnicity. Conducting a multi-center trial is difficult considering that ENB is not yet widely utilized in Asian countries and the investigator-initiated nature of this trial without an external profit-related corporate sponsor. To address this limitation, we planned to enroll eligible participants by over 10 independent pulmonary physicians who will be blinded to the enrollments from other physicians. The ENB procedure will also be performed independently by various physicians. Additionally, the use of multiple biopsy tools in each participant will preclude the specific analysis of procedure-related complication rates associated with individual tools. The design prioritizes evaluation of the possible synergy and differences of biopsy forceps and aspirating needle used in ENB-guided biopsy.

In conclusion, the CONFIDENT-ENB trial is a unique, investigator-initiated trial aimed to identify the optimal tool-selection strategy in ENB-guided lung lesion biopsy, particularly in a least-invasive setting. This study should provide answer to whether the combined use of the two modalities provides synergistic benefits and improve our understanding of the differences between TBNA and forceps biopsy. The CONFIDENT-ENB trial will have a substantial impact on both pulmonology and thoracic oncology specialties and will provide important information regarding the optimal profile of the utility of ENB. Additionally, outcomes of this study will be instructive in designing future comparative studies in the field of guided bronchoscopy in diagnosing lung lesions.

## Data Availability

The datasets generated and/or analyzed during the current study will be available from the corresponding author on reasonable request.

## Abbreviations

CT: Computed tomography
TTNA: Transthoracic needle aspiration
ENB: Electromagnetic navigational bronchoscopy
CONFIDENT-ENB: COmparison and synergistic evaluation of Needle aspiration and Forceps bIopsy for Diagnosing pulmonary lEsioNs wiTh Electromagnetic Navigation Bronchoscopy
BAL: Bronchoalveolar lavage
EBUS: Endobronchial ultrasonography
ROSE: Rapid on-site examination
CTCAE: Common terminology criteria for adverse events
SD: Standard deviation
TBNA: Transbronchial needle aspiration

## References

[1] Bray F, Ferlay J, Soerjomataram I, Siegel RL, Torre LA, Jemal A (2018). Global cancer statistics 2018: GLOBOCAN estimates of incidence and mortality worldwide for 36 cancers in 185 countries. CA Cancer J Clin.

[2] Mazzone PJ, Silvestri GA, Patel S, Kanne JP, Kinsinger LS, Wiener RS (2018). Screening for lung cancer: CHEST guideline and expert panel report. Chest.

[3] McWilliams A, Tammemagi MC, Mayo JR, Roberts H, Liu G, Soghrati K (2013). Probability of cancer in pulmonary nodules detected on first screening CT. N Engl J Med.

[4] Rivera MP, Mehta AC, Wahidi MM (2013). Establishing the diagnosis of lung cancer: Diagnosis and management of lung cancer, 3rd ed: American college of chest physicians evidence-based clinical practice guidelines. Chest.

[5] Silvestri GA, Gonzalez AV, Jantz MA, Margolis ML, Gould MK, Tanoue LT (2013). Methods for staging non-small cell lung cancer: diagnosis and management of lung cancer, 3rd ed: American college of chest physicians evidence-based clinical practice guidelines. Chest.

[6] Gould MK, Donington J, Lynch WR, Mazzone PJ, Midthun DE, Naidich DP (2013). Evaluation of individuals with pulmonary nodules: when is it lung cancer? Diagnosis and management of lung cancer, 3rd ed: American college of chest physicians evidence-based clinical practice guidelines. Chest.

[7] Natioinal comprehensive cancer network. NCCN Guildelines. Non-Small cell lung cancer. Version 5.2021. https://www.nccn.org/professionals/physician_gls/pdf/nscl.pdf.

[8] DiBardino DM, Yarmus LB, Semaan RW (2015). Transthoracic needle biopsy of the lung. J Thorac Dis.

[9] Heerink WJ, de Bock GH, de Jonge GJ, Groen HJ, Vliegenthart R, Oudkerk M (2017). Complication rates of CT-guided transthoracic lung biopsy: meta-analysis. Eur Radiol.

[10] Gasparini S, Mei F, Bonifazi M, Zuccatosta L (2022). Bronchoscopic diagnosis of peripheral lung lesions. Curr Opin Pulm Med.

[11] Folch EE, Pritchett MA, Nead MA, Bowling MR, Murgu SD, Krimsky WS (2019). Electromagnetic Navigation bronchoscopy for peripheral pulmonary lesions: one-year results of the Prospective. Multicenter Navigate Study J Thorac Oncol.

[12] Folch EE, Labarca G, Ospina-Delgado D, Kheir F, Majid A, Khandhar SJ, Mehta HJ (2020). Sensitivity and safety of electromagnetic navigation bronchoscopy for lung cancer diagnosis: systematic review and meta-analysis. Chest.

[13] McGuire AL, Myers R, Grant K, Lam S, Yee J (2020). The diagnostic accuracy and sensitivity for malignancy of radial-endobronchial ultrasound and electromagnetic navigation bronchoscopy for sampling of peripheral pulmonary lesions: systematic review and meta-analysis. J Bronchology Interv Pulmonol.

[14] Gildea TR, Folch EE, Khandhar SJ, Pritchett MA, LeMense GP, Linden PA (2021). The impact of biopsy tool choice and rapid on-site evaluation on diagnostic accuracy for malignant lesions in the prospective: multicenter navigate study. J Bronchology Interv Pulmonol.

[15] Katis K, Inglesos E, Zachariadis E, Palamidas P, Paraskevopoulos I, Sideris G (1995). The role of transbronchial needle aspiration in the diagnosis of peripheral lung masses or nodules. Eur Respir J.

[16] Ost DE, Ernst A, Lei X, Kovitz KL, Benzaquen S, Diaz Mendoza J, Greenhill S, Toth J, Feller Kopman D, Puchalski J, Baram D (2016). Diagnostic yield and complications of bronchoscopy for peripheral lung lesions Results of the AQuIRE registry. Am J Resp Cri Care Med.

[17] Schreiber G, McCrory DC (2003). Performance characteristics of different modalities for diagnosis of suspected lung cancer: summary of published evidence. Chest.

[18] Asano F, Shinagawa N, Ishida T, Shindoh J, Anzai M, Tsuzuku A (2013). Virtual bronchoscopic navigation combined with ultrathin bronchoscopy. A randomized clinical trial. Am J Respir Crit Care Med.

[19] Eberhardt R, Morgan RK, Ernst A, Beyer T, Herth FJ (2010). Comparison of suction catheter versus forceps biopsy for sampling of solitary pulmonary nodules guided by electromagnetic navigational bronchoscopy. Respiration.

[20] Mondoni M, Sotgiu G, Bonifazi M, Dore S, Parazzini EM, Carlucci P (2016). Transbronchial needle aspiration in peripheral pulmonary lesions: a systematic review and meta-analysis. Eur Respir J.

[21] Odronic SI, Gildea TR, Chute DJ (2014). Electromagnetic navigation bronchoscopy-guided fine needle aspiration for the diagnosis of lung lesions. Diagn Cytopathol.

[22] Asahina H, Yamazaki K, Onodera Y, Kikuchi E, Shinagawa N, Asano F (2005). Transbronchial biopsy using endobronchial ultrasonography with a guide sheath and virtual bronchoscopic navigation. Chest.

[23] Eberhardt R, Anantham D, Ernst A, Feller-Kopman D, Herth F (2007). Multimodality bronchoscopic diagnosis of peripheral lung lesions: a randomized controlled trial. Am J Respir Crit Care Med.

[24] Makris D, Scherpereel A, Leroy S, Bouchindhomme B, Faivre JB, Remy J (2007). Electromagnetic navigation diagnostic bronchoscopy for small peripheral lung lesions. Eur Respir J.

[25] Andersen FD, Degn KB, Riis Rasmussen T (2020). Electromagnetic navigation bronchoscopy for lung nodule evaluation. Patient selection, diagnostic variables and safety. Clin Respir J.

[26] National Cancer Institute. Cancer Therapy evaluation program common terminology Criteria for adverse events (CTCAE) v5.0. 2017. Available at: https://ctep.cancer.gov/protocoldevelopment/electronic_applications/ctc.htm.

[27] Wiener RS, Wiener DC, Gould MK (2013). Risks of transthoracic needle biopsy: how high?. Clin Pulm Med.

[28] Hong H, Hahn S, Matsuguma H, Inoue M, Shintani Y, Honda O (2021). Pleural recurrence after transthoracic needle lung biopsy in stage I lung cancer: a systematic review and individual patient-level meta-analysis. Thorax.

[29] Wang Memoli JS, Nietert PJ, Silvestri GA (2012). Meta-analysis of guided bronchoscopy for the evaluation of the pulmonary nodule. Chest.

[30] Steinfort DP, Bonney A, See K, Irving LB (2016). Sequential multimodality bronchoscopic investigation of peripheral pulmonary lesions. Eur Respir J.

[31] Mehta AC, Hood KL, Schwarz Y, Solomon SB (2018). The evolutional history of electromagnetic navigation bronchoscopy: state of the Art. Chest.

[32] Gex G, Pralong JA, Combescure C, Seijo L, Rochat T, Soccal PM (2014). Diagnostic yield and safety of electromagnetic navigation bronchoscopy for lung nodules: a systematic review and meta-analysis. Respiration.

[33] Kim YW, Kim HJ, Song MJ, Kwon BS, Lim SY, Lee YJ (2022). Utility and safety of sole electromagnetic navigation bronchoscopy under moderate sedation for lung cancer diagnosis. Transl Lung Cancer Res.

[34] Chao TY, Chien MT, Lie CH, Chung YH, Wang JL, Lin MC (2009). Endobronchial ultrasonography-guided transbronchial needle aspiration increases the diagnostic yield of peripheral pulmonary lesions: a randomized trial. Chest.

